# Supplementary material for: Dynamics of a methanol-fed marine denitrifying biofilm: 2—impact of environmental changes on the microbial community
Source: PeerJ. 2019 Aug 13;7:e7467. doi: 10.7717/peerj.7467 (PMC6697039; doi:10.7717/peerj.7467)
Supplement: Tables S2–S3 [file peerj-07-7467-s002.docx]

**Supplemental information**

**Table S2 and Figure S2A.**

In the OB, cluster 1 contains 12 OTUs that are most related to *H. nitrativorans*. Sequence identity of these OTUs ranges from 95 to 100%, and composes 92.3% of the *Hyphomicrobium*-affiliated reads. Among these 12 OTU, OTU004 is 100% identical to *H. nitrativorans* and composes 65.1% of the *Hyphomicrobium*-affiliated reads. Cluster 2 comprises five OTUs (6.7% sequences) that are affiliated to *H. vulgare* or *Pedomicrobium australicum*. Finally, three OTUs composed Cluster 3 (1% sequences) that are associated to unclassified *Hyphomicrobiaceae*. In the IO biofilm cultures, the proportion of Cluster 3 is 97.8%, whereas the Clusters 2 and 3 represent 2.0% and 0.2%, respectively. The proportions of the OTUs in Cluster 1 are similar between the OB and the IO biofilm cultures despite the 5.5 fold decrease of proportion of *Hyphomicrobium*-affiliated reads in the IO biofilm cultures.

**Table S3 and Figure S2B.**

Three clusters of *Methylophaga*-affiliated OTUs were derived from the combination of sequences retrieved from OB and IO biofilm cultures. In the OB, cluster 1 contains 11 OTUs that are most related to *M. nitratireducenticrescens,* with sequence identity ranging from 95 to 100% and composes 96.4% of the *Methylophaga*-affiliated reads. Among these OTUs, OTU005 and OTU826 are nearly identical (99.2 to 100%) to *M. nitratireducenticrescens* and comprise 94.3% of the *Methylophaga*-affiliated reads. The two other clusters represent 2.6 and 1%, respectively of the *Methylophaga*-affiliated reads in OB. In IO biofilm cultures, the proportion of overall *Methylophaga*-affiliated reads, increase by 12.2, and the proportion of reads affiliated to Clusters 1 represents 99.7%.

All these results suggest that the *Hyphomicrobium* spp. and *Methylophaga* spp. in the biofilm are a mosaic population of these species with high proportions of *H. nitrativorans* and *M. nitratireducenticrescens.* Because less reads were provided by the pyrosequencing technology, and not the same 16S rRNA regions were sequenced, we could not make a comprehensive analysis of the *Hyphomicrobium* and *Methylophaga*-related sequences from the other biofilm cultures as we did with the OB and IO biofilm cultures.

**Table S2: *Hyphomicrobium*-affiliated OTUs in the OB and the IO biofilm cultures**

Sequence identity with Number reads Proportion of reads %

*H. nitrativorans* OB IO OB IO

Cluster 1 95-100% Total 147209 26239 92.3 97.8

OTU004 107700 19419

OTU257 22538 4232

OTU106 195 20

OTU214 7237 1366

OTU357 221 17

OTU285 64 0

OTU123 2481 217

OTU713 1288 2

OTU147 70 21

OTU706 4993 819

OTU848 383 126

OTU329 39 0

Cluster 2 91-97% Total 10779 530 6.8 2.0

OTU980 23 0

OTU010 5273 29

OTU102 202 0

OTU353 17 0

OTU212 5264 501

Cluster 3 89-92% Total 1537 51 0.96 0.19

OTU029 1501 1

OTU375 21 0

OTU274 15 50

16S rRNA gene sequences retrieved from total DNA of the OB and the IO biofilm cultures and affiliated to the *Hyphomicrobium* spp. were aligned and clustered based on 97% identity. Representative sequences from the resulting OTUs were realigned and a phylogenic analysis were performed (see Figure S2A for details).

**Table S3: *Methylophaga*-affiliated OTUs in the OB and the IO biofilm cultures**

Sequence identity with Number reads Proportion of reads %

*M. nitratireducenticrescens* OB IO OB IO

Cluster 1 94-100% Total 11809 136197 96.4 99.7

OTU5 10486 120034

OTU826 1055 11840

OTU258 139 1817

OTU408 0 55

OTU645 19 78

OTU475 9 52

OTU181 52 85

OTU128 12 211

OTU85 0 277

OTU247 0 38

OTU299 0 130

OTU593 0 35

OTU538 0 20

OTU273 3 219

OTU101 2 1069

OTU313 9 212

OTU938 0 24

OTU282 23 1

Cluster 2 92.9-93.6% Total 321 284 2.62 0.21

OTU68 1 283

OTU634 110 1

OTU76 210 0

Cluster 3 93.4-94.1% Total 115 65 0.94 0.05

OTU159 115 4

OTU206 0 61

16S rRNA gene sequences retrieved from total DNA of the OB and the IO biofilm cultures and affiliated to the *Methylophaga* spp. were aligned and clustered based on 97% identity. Representative sequences from the resulting OTUs were realigned and a phylogenic analysis were performed (see Figure S2B for details).
