# Supplementary material for: Dynamics of a methanol-fed marine denitrifying biofilm: 2—impact of environmental changes on the microbial community
Source: PeerJ. 2019 Aug 13;7:e7467. doi: 10.7717/peerj.7467 (PMC6697039; doi:10.7717/peerj.7467)
Supplement: Figure S1 [file peerj-07-7467-s003.pdf]

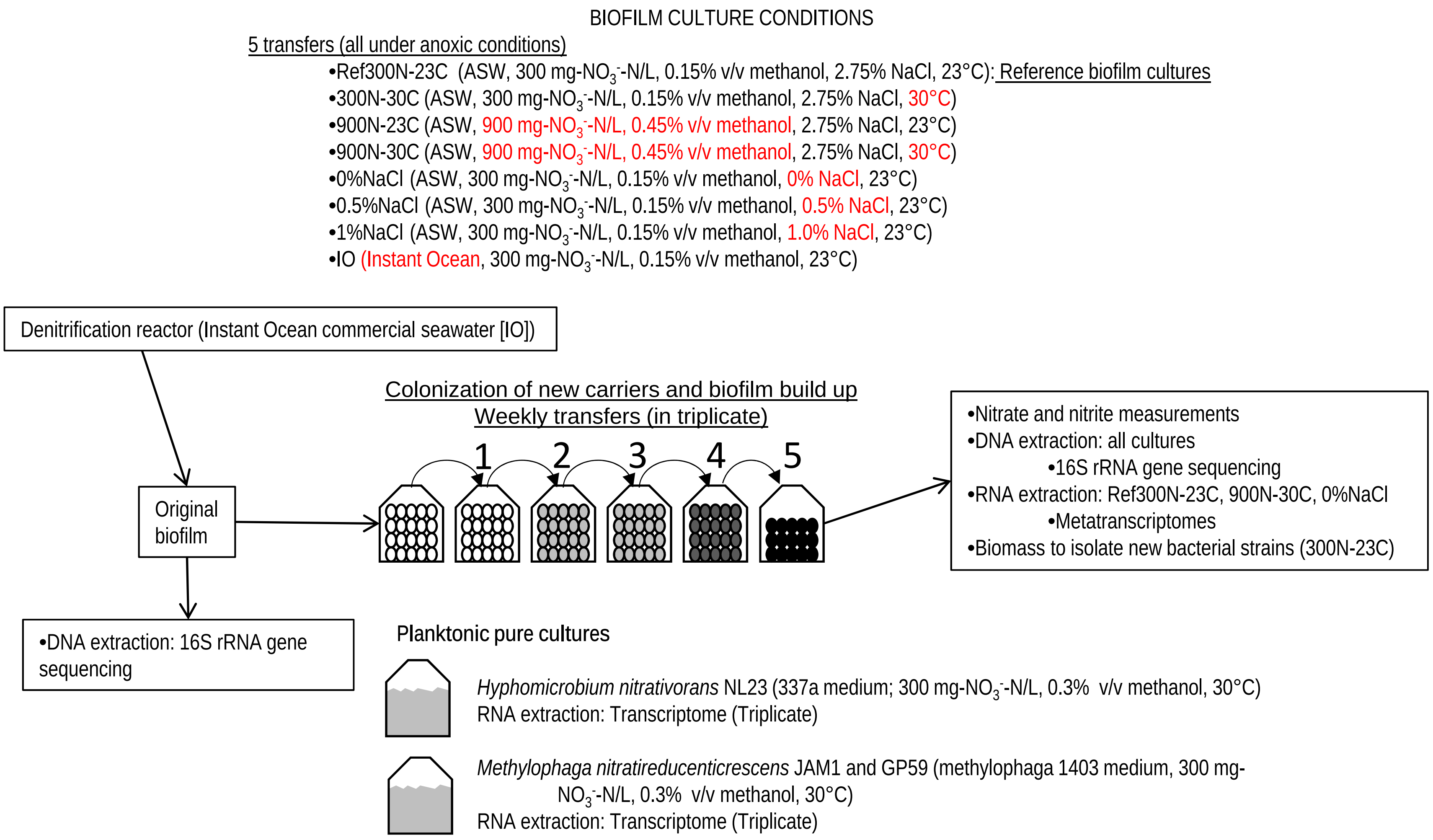

Figure S1. Schematic of the experimental assays performed in the study

The original biofilm was thawed, scraped from the carriers, dispersed and distributed in vials containing the ASW medium supplemented with prescribed concentrations of NO_3^-, methanol and NaCl, or containing the IO medium (Table 1), and 20 free carriers. The vials were incubated at the prescribed temperature under anoxic conditions. The carriers were transferred five times at appr. 1 week interval into fresh prescribed medium and incubated in the same conditions. NO_3^- and NO_2^- concentrations were measured every 24-48 h. Methanol and $NaNO_3$ were added when needed if NO_3^- was completely depleted between transfers. DNA or RNA was extracted from the biofilm at the end of the 5th transfer cultures. Planktonic pure cultures were carried out in recommended culture medium for the respective strains.
