# Supplementary material for: Dynamics of a methanol-fed marine denitrifying biofilm: 2—impact of environmental changes on the microbial community
Source: PeerJ. 2019 Aug 13;7:e7467. doi: 10.7717/peerj.7467 (PMC6697039; doi:10.7717/peerj.7467)
Supplement: Figure S2 [file peerj-07-7467-s004.pdf]

Figure S2. Evolutionary relationships of OTUs derived from the OB and the IO biofilm cultures with the genera *Hyphomicrobium* and *Methylophaga*

An unrooted phylogenetic tree demonstrating the evolutionary relationship of 16S rRNA gene sequences (V6-V7-V8 regions) is illustrated. The evolutionary history was inferred using the Minimum Evolution method [1]. The percentage of replicate trees in which the associated taxa clustered together in the bootstrap test (1000 replicates) are shown next to the branches [2]. The tree is drawn to scale, with branch lengths in the same units as those of the evolutionary distances used to infer the phylogenetic tree. The evolutionary distances were computed using the Maximum Composite Likelihood method [3] and are in the units of the number of base substitutions per site. The rate variation among sites was modeled with a gamma distribution (shape parameter = 1.7). The ME tree was searched using the Close;Neighbor;Interchange (CNI) algorithm [4] at a search level of 1. The Neighbor;joining algorithm [5] was used to generate the initial tree. All positions with less than 95% site coverage were eliminated. That is, fewer than 5% alignment gaps, missing data, and ambiguous bases were allowed at any position. There were a total of 409 positions in the final dataset. Evolutionary analyses were conducted in MEGA6 [6]. Beside OTU names are the number of reads derived from the OB and the IO biofilm cultures, respectively, and the percentage of identity with *H. nitrativorans* NL23 16S sequence (Panel A) or with *M. nitratireducenticrescens* JAM1 16S sequence (Panel B). Beside the species names is the GenBank accession number.

Panel A: *Hyphomicrobium*
The optimal tree with the sum of branch length = 1.70056439 is shown. The analysis involved 46 nucleotide sequences.

Panel B: *Methylophaga*
The optimal tree with the sum of branch length = 1.56790826 is shown. The analysis involved 54 nucleotide sequences.

1. Rzhetsky A. and Nei M. (**1992**). A simple method for estimating and testing minimum evolution trees. *Molecular Biology and Evolution* **9**:945-967.
2. Felsenstein J. (**1985**). Confidence limits on phylogenies: An approach using the bootstrap. *Evolution* **39**:783-791.
3. Tamura K., Nei M., and Kumar S. (**2004**). Prospects for inferring very large phylogenies by using the neighbor-joining method. *Proceedings of the National Academy of Sciences* (*USA*) **101**:11030-11035.
4. Nei M. and Kumar S. (**2000**). *Molecular Evolution and Phylogenetics*. Oxford University Press, New York.
5. Saitou N. and Nei M. (**1987**). The neighbor-joining method: A new method for reconstructing phylogenetic trees. *Molecular Biology and Evolution* **4**:406-425.
6. Tamura K., Stecher G., Peterson D., Filipski A., and Kumar S. (**2013**). MEGA6: Molecular Evolutionary Genetics Analysis version 6.0. *Molecular Biology and Evolution*30: 2725-2729.

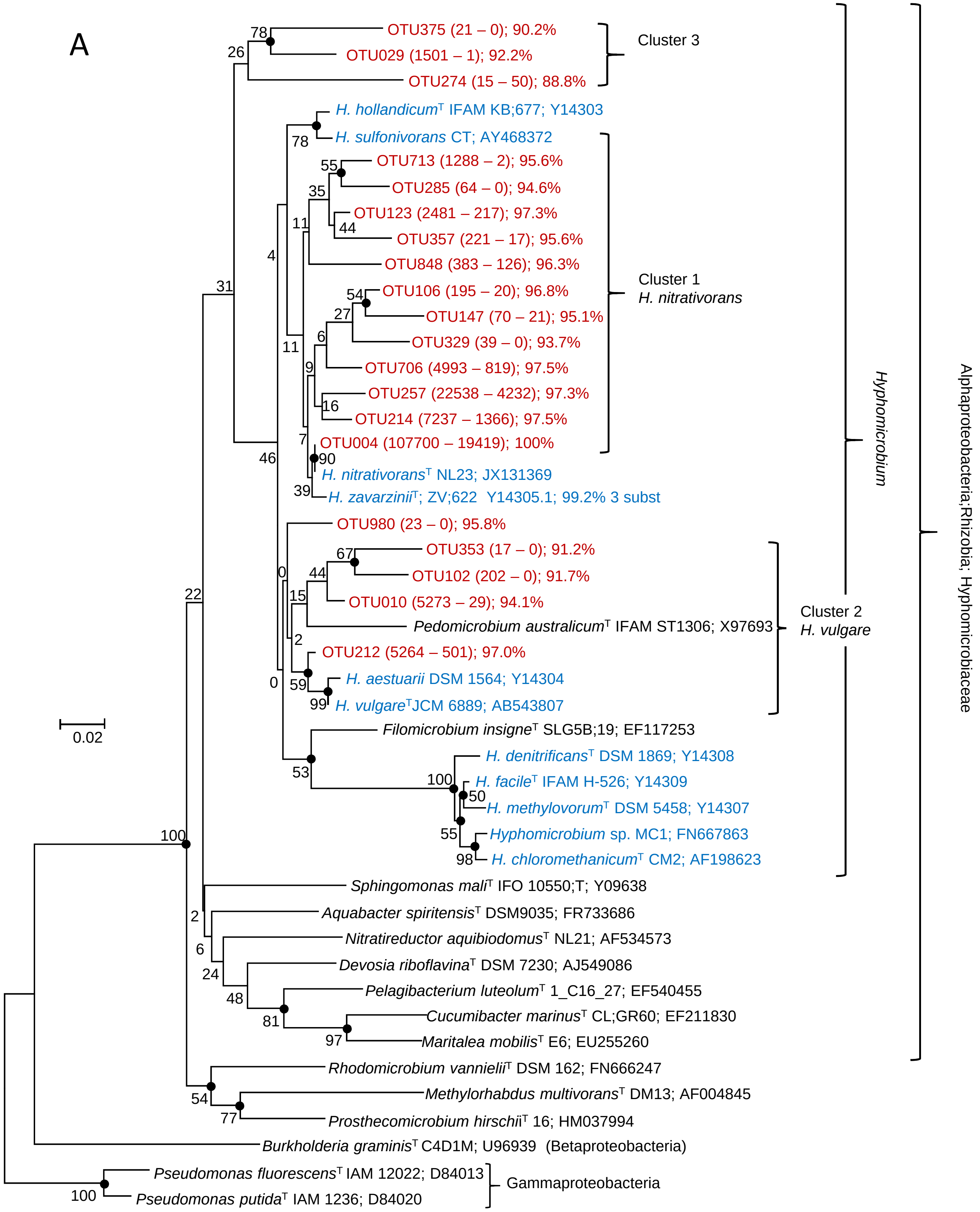
A
OTU375 (21 – 0); 90.2%
OTU029 (1501 – 1); 92.2%
OTU274 (15 – 50); 88.8%
Cluster 3
H. hollandicum^T IFAM KB;677; Y14303
H. sulfonivorans CT; AY468372
OTU713 (1288 – 2); 95.6%
OTU285 (64 – 0); 94.6%
OTU123 (2481 – 217); 97.3%
OTU357 (221 – 17); 95.6%
OTU848 (383 – 126); 96.3%
OTU106 (195 – 20); 96.8%
OTU147 (70 – 21); 95.1%
OTU329 (39 – 0); 93.7%
OTU706 (4993 – 819); 97.5%
OTU257 (22538 – 4232); 97.3%
OTU214 (7237 – 1366); 97.5%
OTU004 (107700 – 19419); 100%
Cluster 1
H. nitrativorans
H. nitrativorans^T NL23; JX131369
H. zavarzinii^T; ZV;622 Y14305.1; 99.2% 3 subst
OTU980 (23 – 0); 95.8%
OTU353 (17 – 0); 91.2%
OTU102 (202 – 0); 91.7%
OTU010 (5273 – 29); 94.1%
Pedomicrobium australicum^T IFAM ST1306; X97693
OTU212 (5264 – 501); 97.0%
H. aestuarii DSM 1564; Y14304
H. vulgare^T JCM 6889; AB543807
Cluster 2
H. vulgare
Filomicrobium insigne^T SLG5B;19; EF117253
H. denitrificans^T DSM 1869; Y14308
H. facile^T IFAM H-526; Y14309
H. methylovorum^T DSM 5458; Y14307
Hyphomicrobium sp. MC1; FN667863
H. chloromethanicum^T CM2; AF198623
Hyphomicrobium
Alphaproteobacteria;Rhizobia; Hyphomicrobiaceae
Sphingomonas mali^T IFO 10550;T; Y09638
Aquabacter spiritensis^T DSM9035; FR733686
Nitratireductor aquibiodomus^T NL21; AF534573
Devosia riboflavina^T DSM 7230; AJ549086
Pelagibacterium luteolum^T 1_C16_27; EF540455
Cucumibacter marinus^T CL;GR60; EF211830
Maritalea mobilis^T E6; EU255260
Rhodomicrobium vannielii^T DSM 162; FN666247
Methylorhabdus multivorans^T DM13; AF004845
Prosthecomicrobium hirschii^T 16; HM037994
Burkholderia graminis^T C4D1M; U96939 (Betaproteobacteria)
Pseudomonas fluorescens^T IAM 12022; D84013
Pseudomonas putida^T IAM 1236; D84020
Gammaproteobacteria
0.02
78
26
78
55
35
44
11
4
54
27
6
11
9
16
31
7
90
46
39
67
44
0
15
2
22
0
59
99
53
100
50
55
98
100
2
6
24
48
81
97
54
77
100

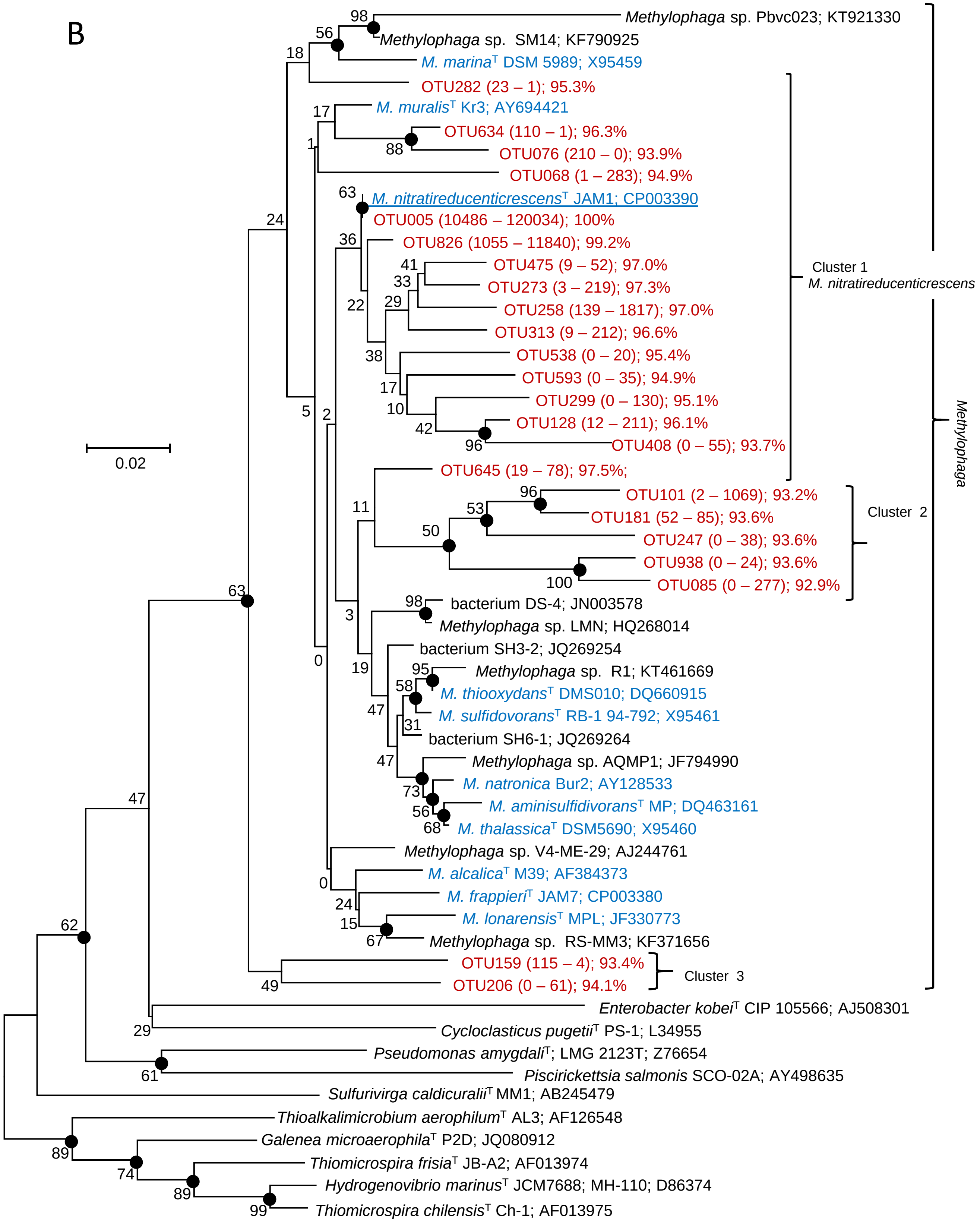

B
Methylophaga sp. Pbvc023; KT921330
Methylophaga sp. SM14; KF790925
M. marina T DSM 5989; X95459
OTU282 (23 – 1); 95.3%
M. muralis T Kr3; AY694421
OTU634 (110 – 1); 96.3%
OTU076 (210 – 0); 93.9%
OTU068 (1 – 283); 94.9%
M. nitratireducenticrescens T JAM1; CP003390
OTU005 (10486 – 120034); 100%
OTU826 (1055 – 11840); 99.2%
OTU475 (9 – 52); 97.0%
OTU273 (3 – 219); 97.3%
OTU258 (139 – 1817); 97.0%
OTU313 (9 – 212); 96.6%
OTU538 (0 – 20); 95.4%
OTU593 (0 – 35); 94.9%
OTU299 (0 – 130); 95.1%
OTU128 (12 – 211); 96.1%
OTU408 (0 – 55); 93.7%
Cluster 1
M. nitratireducenticrescens
Methylophaga
0.02
OTU645 (19 – 78); 97.5%;
OTU101 (2 – 1069); 93.2%
OTU181 (52 – 85); 93.6%
OTU247 (0 – 38); 93.6%
OTU938 (0 – 24); 93.6%
OTU085 (0 – 277); 92.9%
Cluster 2
bacterium DS-4; JN003578
Methylophaga sp. LMN; HQ268014
bacterium SH3-2; JQ269254
Methylophaga sp. R1; KT461669
M. thiooxydans T DMS010; DQ660915
M. sulfidovorans T RB-1 94-792; X95461
bacterium SH6-1; JQ269264
Methylophaga sp. AQMP1; JF794990
M. natronica Bur2; AY128533
M. aminisulfidivorans T MP; DQ463161
M. thalassica T DSM5690; X95460
Methylophaga sp. V4-ME-29; AJ244761
M. alcalica T M39; AF384373
M. frappieri T JAM7; CP003380
M. lonarensis T MPL; JF330773
Methylophaga sp. RS-MM3; KF371656
OTU159 (115 – 4); 93.4%
OTU206 (0 – 61); 94.1%
Cluster 3
Enterobacter kobei T CIP 105566; AJ508301
Cycloclasticus pugetii T PS-1; L34955
Pseudomonas amygdali T; LMG 2123T; Z76654
Piscirickettsia salmonis SCO-02A; AY498635
Sulfurivirga caldicuralii T MM1; AB245479
Thioalkalimicrobium aerophilum T AL3; AF126548
Galenea microaerophila T P2D; JQ080912
Thiomicrospira frisia T JB-A2; AF013974
Hydrogenovibrio marinus T JCM7688; MH-110; D86374
Thiomicrospira chilensis T Ch-1; AF013975
