# Supplementary material for: Dynamics of a methanol-fed marine denitrifying biofilm: 2—impact of environmental changes on the microbial community
Source: PeerJ. 2019 Aug 13;7:e7467. doi: 10.7717/peerj.7467 (PMC6697039; doi:10.7717/peerj.7467)
Supplement: Figures S3–S4 [file peerj-07-7467-s005.pdf]

Figure S3. Relative expression profiles of *M. nitratireducenticrescens* strain GP59 in Ref300N-23C and 900N-30C biofilm cultures

All the deduced amino acid sequences associated to the GP59 genome and plasmids in the Ref300N-23C and 900N-30C biofilm cultures were submitted to the BlastKOALA (genome annotation and KEGG mapping) at the Kyoto encyclopedia of genes and genomes (KEGG). Genes associated to specific metabolisms were sorted out and the corresponding ratio of the Biofilm TPM versus the pure culture TPM was derived. When the ratios were <1, the negative inverse value (-1/ratio) was calculated. Data are expressed as the percentage of genes in each category that are more expressed in the biofilm cultures (right, 2 to 5 times, and > 5 times) or in pure cultures (left, -2 to -5 times and > -5 times). Number within parentheses is the number of genes involved in the selected pathways. (see Supplemental document 8).

Figure S4. Relative expression profiles of *M. nitratireducenticrescens* strain GP59 and *H. nitrativorans* Nl23 in the 0%NaCl biofilm cultures

All the deduced amino acid sequences associated to the GP59 genome and plasmids and the NL23 genome in the 0%NaCl biofilm cultures were analyzed as described in Figure legend S3 (see Supplemental document 8).

Relative expression profiles of *M. nitratireducenticrescens* strain JAM1 are detailed in the Supplemental documents 8 and 11.

Figure S3

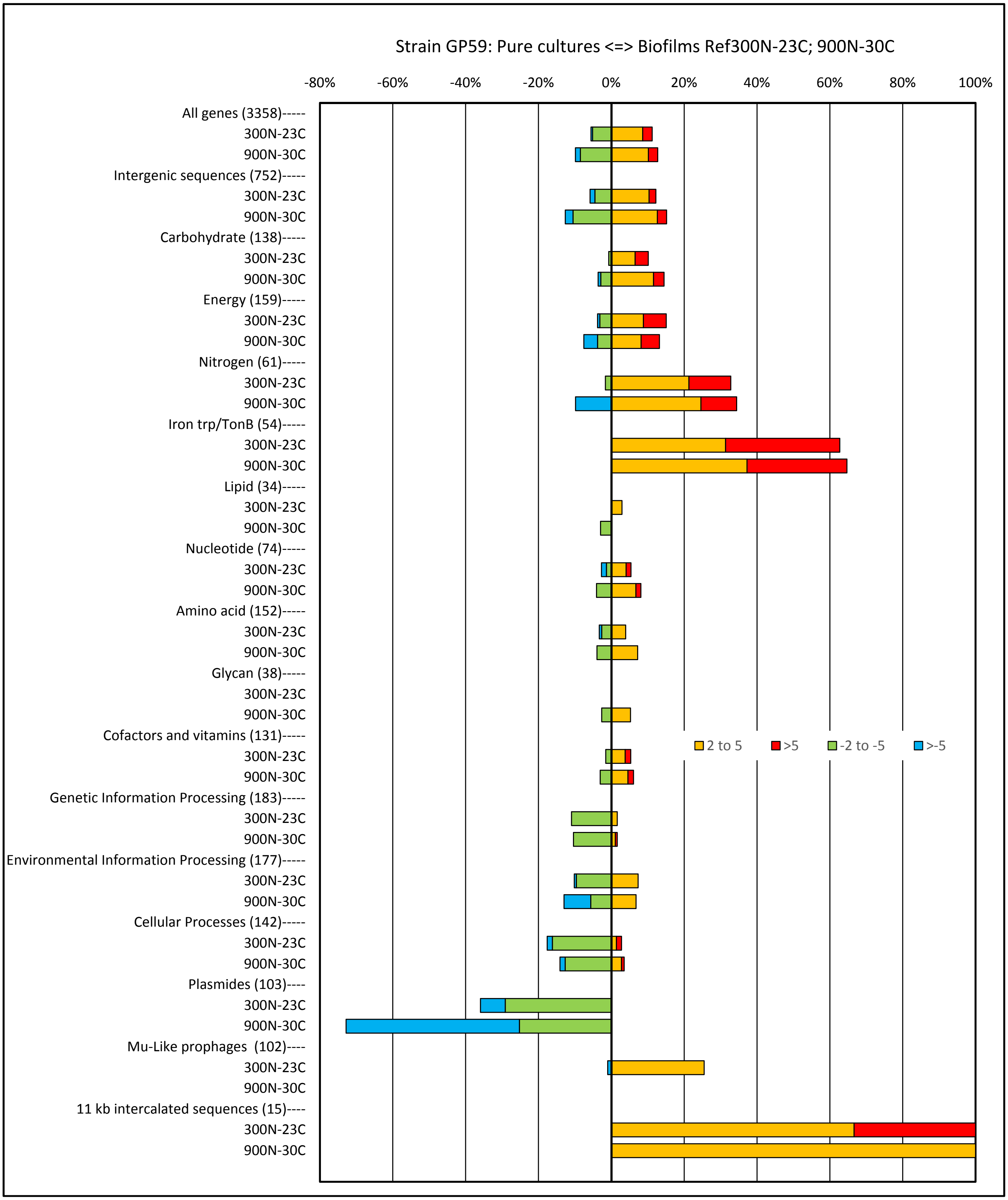

Strain GP59: Pure cultures <=> Biofilms Ref300N-23C; 900N-30C
-80%
-60%
-40%
-20%
0%
20%
40%
60%
80%
100%
All genes (3358)-----
300N-23C
900N-30C
Intergenic sequences (752)-----
300N-23C
900N-30C
Carbohydrate (138)-----
300N-23C
900N-30C
Energy (159)-----
300N-23C
900N-30C
Nitrogen (61)-----
300N-23C
900N-30C
Iron trp/TonB (54)-----
300N-23C
900N-30C
Lipid (34)-----
300N-23C
900N-30C
Nucleotide (74)-----
300N-23C
900N-30C
Amino acid (152)-----
300N-23C
900N-30C
Glycan (38)-----
300N-23C
900N-30C
Cofactors and vitamins (131)-----
300N-23C
900N-30C
Genetic Information Processing (183)-----
300N-23C
900N-30C
Environmental Information Processing (177)-----
300N-23C
900N-30C
Cellular Processes (142)-----
300N-23C
900N-30C
Plasmides (103)----
300N-23C
900N-30C
Mu-Like prophages (102)----
300N-23C
900N-30C
11 kb intercalated sequences (15)----
300N-23C
900N-30C
2 to 5
>5
-2 to -5
>-5

Figure S4

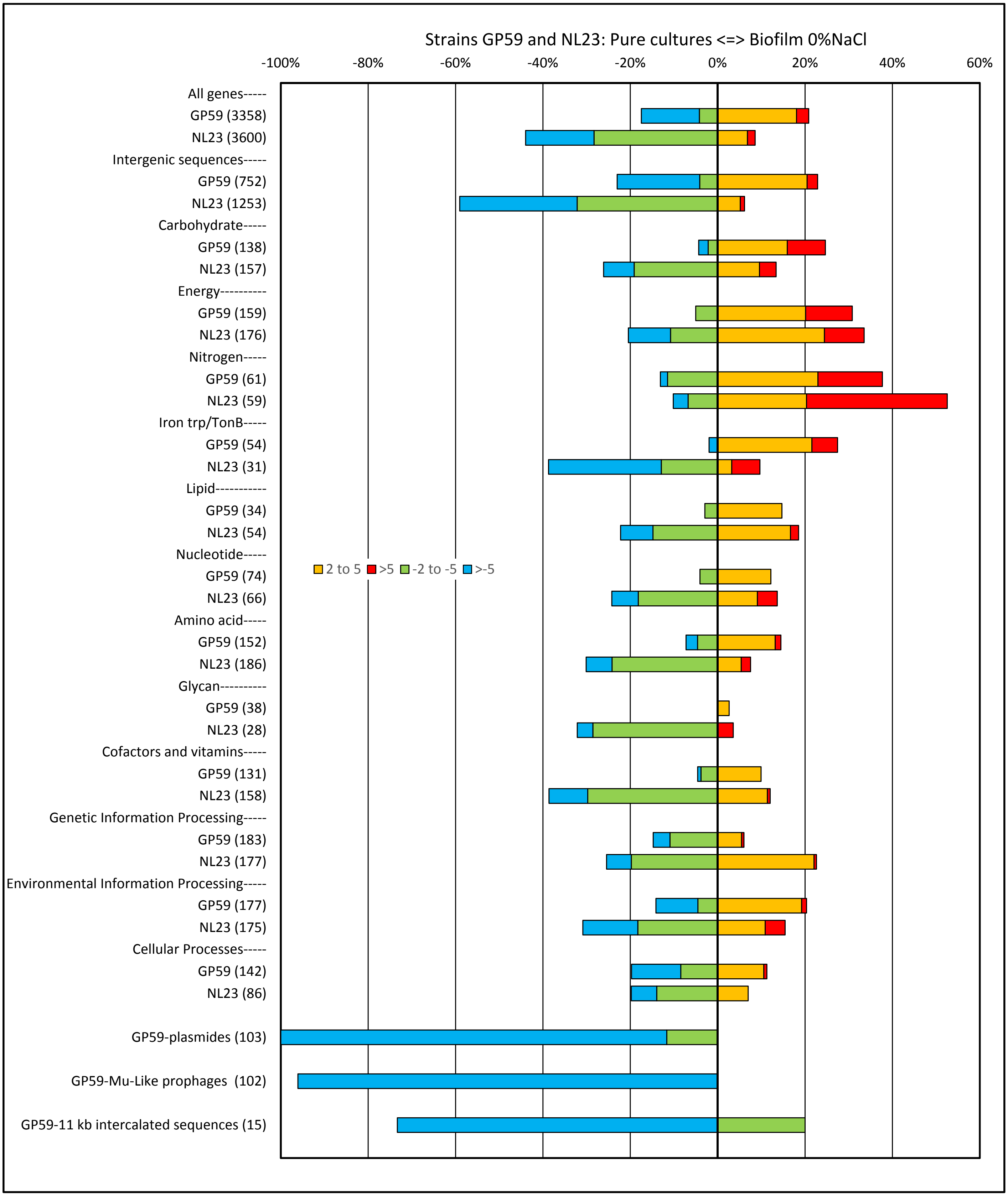
Strains GP59 and NL23: Pure cultures <=> Biofilm 0%NaCl
-100%
-80%
-60%
-40%
-20%
0%
20%
40%
60%
All genes-----
GP59 (3358)
NL23 (3600)
Intergenic sequences-----
GP59 (752)
NL23 (1253)
Carbohydrate-----
GP59 (138)
NL23 (157)
Energy----------
GP59 (159)
NL23 (176)
Nitrogen-----
GP59 (61)
NL23 (59)
Iron trp/TonB-----
GP59 (54)
NL23 (31)
Lipid-----------
GP59 (34)
NL23 (54)
Nucleotide-----
GP59 (74)
NL23 (66)
Amino acid-----
GP59 (152)
NL23 (186)
Glycan----------
GP59 (38)
NL23 (28)
Cofactors and vitamins-----
GP59 (131)
NL23 (158)
Genetic Information Processing-----
GP59 (183)
NL23 (177)
Environmental Information Processing-----
GP59 (177)
NL23 (175)
Cellular Processes-----
GP59 (142)
NL23 (86)
GP59-plasmides (103)
GP59-Mu-Like prophages (102)
GP59-11 kb intercalated sequences (15)
2 to 5
>5
-2 to -5
>-5
