# Supplementary material for: Dynamics of a methanol-fed marine denitrifying biofilm: 2—impact of environmental changes on the microbial community
Source: PeerJ. 2019 Aug 13;7:e7467. doi: 10.7717/peerj.7467 (PMC6697039; doi:10.7717/peerj.7467)
Supplement: Supplemental Information 1 — Brief description of: the 14 supplemental documents, the accession numbers of the 16S rRNA sequences and transcriptomes, and where they were used to derive the Figures, Tables and supplemental files. [file peerj-07-7467-s006.docx]

Supplemental document 1

RTL-Data_Analysis_Methodology: procedure used by the RTL sequencing service to process the 16S rRNA sequence reads.

Supplemental document 2

- Raw data for Figure 1 and Table 2
- OTU sequences

Supplemental document 3

- Metatranscriptome analysis
  - - Reference genome: strain GP59 Biofilm cultures-vs-pure cultures
    - Reference genome: strain NL23 Biofilm cultures-vs-pure cultures
    - Reference genome: strain JAM1 Biofilm cultures-vs-pure cultures

Supplemental document 4

- Raw data for Table 6

Supplemental document 5

- Analysis of de novo assembled transcripts of the Ref300N-23C biofilm cultures

Supplemental document 6

- Analysis of de novo assembled transcripts of the 900N-30C biofilm cultures

Supplemental document 7

- Analysis of de novo assembled transcripts of the 0%NaCl biofilm cultures

Supplemental document 8

- Raw data for Figure 2 and Figures S3-S4. Supplemental information regarding the metatranscriptome analysis associated to strain JAM1

Supplemental document 9

- Rawdata for Figure 3A, Table 5 and Figure S3-S4 related to strain GP59

Supplemental document 10

- Rawdata for Figure 3B, Table 5 and Figure S4 related to strain NL23

Supplemental document 11

- Metatranscriptome analysis associated to strain JAM1

Supplemental document 12

- Rawdata for Figure 4

Supplemental document 13

- Aligned sequences for Figure S2A (Fasta format)

Supplemental document 14

- Aligned sequences for Figure S2B (Fasta format)

Material and Methonds

-**Table 1:** Biofilm culture conditions no raw data

-**Figure S1**: Schematic of the experimental assays performed in the study no raw data

-16S rRNA gene sequences:

GenBank, SRA: PRJNA524642

-Complete analyses of the 16S rRNA gene sequences s (affiliation and OTU sequences)

File: Supplemental document 2

-16S 16S rRNA gene sequences for isolates:

GenBank, MK571459 to MK571476.

-Transcriptomes of the:

a) three strains (FastaQ format)

b) biofilm metatranscriptomes (FastaQ format),

c) unaligned assembled de novo transcripts (Fasta format)

GenBank, SRA: PRJNA525230

-Metatranscriptome analyses (from reads to TPM and Ratios)

File: Supplemental document 3

Sheet 1: Strain GP59 pure cultures vs biofilm cultures

Sheet 2: Strain NL23 pure cultures vs biofilm cultures

Sheet 3: Strain JAM1 pure cultures vs biofilm cultures

-The annotated de novo assembled transcripts at JGI: GOLD Analysis Project Id:

Ga0307915, Ref300C-23C biofilm cultures

Ga0307877, 900C-30C biofilm cultures

Ga0307760, 0%NaCl biofilm cultures

-Functional and Microbial Diversity of the de novo assembled transcripts (For Table 6)

Files:

Supplemental document 4 Table 6 de novo Summary

Supplemental document 5 Table 6-Biofilm Ref300N23C-denovo

Supplemental document 6 Table 6-Biofilm 900N30C-denovo

Supplemental document 7 Table 6-Biofilm 0%NaCl-denovo

RESULTS

**-16S rRNA gene sequences sections**

**Figure 1**. Proportion of affiliated OTUs in the biofilm cultures.

Supplemental document 2: Sheet summary

**Table 2**: Most probable affiliation of the most abundant 16S rRNA sequences in the biofilm cultures and the original biofilm

Supplemental document 2: Sheet summary

**Table 3**. OTUs affiliated to *Hyphomicrobium* spp. and *Methylophaga* spp. in the OB and the IO biofilm cultures

Supplemental document 2: Sheets OB and IO

**Figure S2A and B**: Evolutionary relationships of OTUs derived from the OB and the IO biofilm cultures with the genera *Hyphomicrobium* and *Methylophaga*

Rawdata Figure S2A.fas

Rawdata Figure S2B.fas

**Table S2**: *Hyphomicrobium*-affiliated OTUs in the OB and the IO biofilm cultures

Supplemental document 2: Sheets OB and IO

**Table S3:** *Methylophaga*-affiliated OTUs in the OB and the IO biofilm cultures

Supplemental document 2: Sheets OB and IO

**-Isolates**

**Table 4.** Affiliation of the isolates isolated from the reference biofilm cultures No raw data

**Metatranscriptomes**

**Figure 2**. Relative expression profiles of *M. nitratireducenticrescens* GP59 and *H. nitrativorans* NL23 in biofilm cultures

Supplemental document 8 Sheet: Figure 2

**Figure S3.** Relative expression profiles of *M. nitratireducenticrescens* strain GP59 in Ref300N-23C and 900N-30C biofilm cultures

Supplemental document 8

Sheet: Figures S3-S4 (GP59 and NL23 only)

Sheet: 3 strains (JAM1 data included)

**Figure S4.** Relative expression profiles of *M. nitratireducenticrescens* strain GP59 and *H. nitrativorans* Nl23 in the 0%NaCl biofilm cultures

Supplemental document 8

Sheet: Figures S3-S4 (GP59 and NL23 only)

Sheet: 3 strains (JAM1 data included)

**Figure 3A and 3B:** Relative gene expression profiles of selected metabolic pathways in *M.* *nitratireducenticrescens* GP59 and *H. nitrativorans* NL23 in the biofilm cultures

Supplemental document 9, GP59

Supplemental document 10, NL23

-Supplemental document 11

Relative gene expression profiles of selected metabolic pathways in

*M.* *nitratireducenticrescens* JAM1

**Table 5:** Changes in the relative transcript levels of genes involved in the nitrogen metabolism

Supplemental document 9, Sheet: Nitrogen summary

**Table 6:** Microbial diversity and the associated denitrification genes in the biofilm cultures

Supplemental documents 4, 5, 6 and 7

**Figure 4**: Hierarchical clustering of the bacterial populations in biofilm culture metatranscriptomes.

Supplemental document 12: Rawdata Figure 4
