# Supplementary material for: Dynamics of a methanol-fed marine denitrifying biofilm: 2—impact of environmental changes on the microbial community
Source: PeerJ. 2019 Aug 13;7:e7467. doi: 10.7717/peerj.7467 (PMC6697039; doi:10.7717/peerj.7467)
Supplement: Document S1 — Procedures used by the RTL sequencing service to process the 16S rRNA sequence reads. [file peerj-07-7467-s007.pdf]

RTL GENOMICS

A DIVISION OF RESEARCH AND TESTING LABORATORIES

Data Analysis Methodology

Last Updated: 08/01/2016

The most recent version of this file can be downloaded from
http://www.rtlgenomics.com/docs/Data_Analysis_Methodology.pdf

Contents

Version Changelog 4
Current Version 4
Version 2.3.1 (08/01/2016) 4
Previous Versions 4
FAQ (Frequently Asked Questions) 6
What does R1 and R2 mean on my raw FASTQ files? 6
Can I use QIIME or MOTHUR with RTLGenomics Data? 6
How do I use QIIME with RTLGenomics Data? 6
How do I use MOTHUR with RTLGenomics Data? 7
What exactly does *No Hit* mean in my analysis? 7
What is the difference between *Unknown* and *Unclassified* in my analysis files? 8
How are confidence values determined? 8
What is the difference between FullTaxa and TrimmedTaxa Files? 10
Do my sequences contain the primer, barcodes or adapters? 11
Term Definitions 12
Database Maintenance Policy 12
Client Data Retention Policy 12
Data Analysis Methodology 13
Visual Overview of the Data Analysis Process 13
Overview of the Data Analysis Process 13
Denoising and Chimera Checking 14
Denoising 14
Chimera Checking 16
Microbial Diversity Analysis 16
Quality Checking and FASTA Formatted Sequence/Quality File Generation 17
OTU Selection 18

Tree Building 18
Taxonomic Identification 18
Diversity Analysis 19
File Descriptions and Formatting 20
Zip Archives 20
Split Zip Archives 20
Raw Sequence Data File Formats 21
SFF File Generation 21
FASTQ File Generation 21
Roche 454 22
IonTorrent PGM 22
Illumina MiSeq 22
FASTA Archive File Descriptions 23
Analysis Archive File Descriptions 25
Recommended Programs 30
References 32

Version Changelog

Current Version

Version 2.3.1 (08/01/2016)

- Corrected the Paired-End illustrations.
- Updated the FAQ.

Previous Versions

Version 2.3 (04/29/2016)

- Added reverse primer information to the FASTA archive.
- Updated the analysis pipeline graphic.
- Added the database maintenance policy.
- Added the FAQ (Frequently Asked Questions)
- Updated the file descriptions to add the new oligos files we provide.

Version 2.2.4 (11/07/2014)

- Updated the file descriptions to include the file type and recommended program for viewing the data.
- Updated the file descriptions to include all files provided by RTL.
- Added new section to cover recommended programs for viewing provided data.

Version 2.2.3 (09/16/2014)

- Updated the data archive to split files too large to fit on our webserver.
- Included instructions for how to handle split zip archives.

Version 2.2.2 (09/03/2014)

- Added the OTUs folder to the Analysis archive.
- Moved OTUmap.txt from Analysis/OTUMap.txt to Analysis/OTUs/OTUMap.txt
- Added OTUs.fas to the Analysis/OTUs archive.
- Corrected the otus.tre file. All ‘;’ within sequence definitions have been changed to ‘_’.

Version 2.2.1 (08/29/2014)

- Added the customer data retention policy.

Version 2.2.0 (07/09/2014)

- Added phylogenetic tree construction using MUSCLE and FastTree.
- Added Krona visualization to the Taxonomic Analysis pipeline.
- Added phylogenetic tree, multiple sequence alignment, and Krona visualizations to the analysis zip archive.
- Updated 454 and Ion Torrent PGM processing to run using the same workflow as MiSeq.
- Added description for the OTUMap.txt file in the Analysis Folder.

Version 2.1.1 (05/20/2014)

- Updated OTU Selection. Trimming to shortest sequence now performed before UPARSE OTU Selection.

Version 2.1.0 (02/28/2014)

- Updated denoiser to use PEAR for paired-end read merging in place of USEARCH.

Version 2.0.0 (01/28/2014)

- Updated denoiser to use USEARCH 7, replacing USEARCH 5.
- Methodology now accounts for processing of 454, Ion Torrent PGM and Illumina MiSeq data.

FAQ (Frequently Asked Questions)

What does R1 and R2 mean on my raw FASTQ files?

Most sequencing performed on our Illumina sequencers was run using paired-end sequencing. This generates two FASTQ files where the first (R1) file contains reads that start at the forward primer and go towards the reverse and the second (R2) file contains the corresponding reverse reads that are oriented as starting from the reverse primer and going towards the forward. For more information please see the section titled "Paired End FASTQ Files" found below on page 23.

Can I use QIIME or MOTHUR with RTLGenomics Data?

QIIME [1] and MOTHUR [2] are open-source bioinformatics pipelines for performing microbiome analysis from raw DNA sequencing data. RTLGenomics provides clients that receive our analysis a copy of their data already quality checked and ready for use in both QIIME and MOTHUR. You can find the sequence data for QIIME and MOTHUR in the FASTA zip archive discussed in the section "FASTA Archive File Descriptions" found on page 23.

You can find documentation for or download QIIME at http://qiime.org/.

You can find documentation for or download MOTHUR at http://www.mothur.org.

How do I use QIIME with RTLGenomics Data?

QIIME's split_libraries.py workflow is used to demultiplex sequence data and format it to work with the downstream analysis tools. This particular workflow requires three files to work, a FASTA formatted sequence file, a FASTA formatted quality file and a mapping file that tells QIIME what the sample's names, barcodes and primers are. We strongly recommend you use the following options when running split_libraries.py using our FASTA data:

split_libraries.py –m <Mapping TXT file> -f <Fasta FNA File> -q <Qual File> -o <Output Folder> -H 1000 –p

The –m, -f, -q and –o options are all required and tell the workflow where the mapping, FASTA sequence, FASTA quality and output files are respectively. However we also recommend you add the following options:

- –p
    - The –p options instructs QIIME to ignore the primer because otherwise it will require the primer be present in order for the sequence to be valid.

- While the 454 and PGM platforms will sequence the primer, the Illumina platform does not sequence it. It might be present in the raw data if we performed a double method PCR.
- Our pipeline removes the primer from each sequence during our QC steps, so even if the primer exists on the raw data it will not exist in the data that has been prepared for QIIME.

- –H 1000
    - The –H option sets the maximum number of homopolymers the sequence can contain and still be considered valid.
    - We recommend using this option with Illumina data but do not use it with 454 or PGM data.
    - 454 and PGM sequencers suffer from issues when homopolymers longer than 4-6 base pairs are encountered however the Illumina platform does not suffer from issues regarding homopolymers (at least not the point that the other platforms do). As such you can set the –H option to a very high number to keep QIIME from throwing away valid sequences. This is a bigger issue in fungal samples than bacterial.

How do I use MOTHUR with RTLGenomics Data?

MOTHUR's Trim.seqs command is used to demultiplex sequence data and format it to work with the downstream analysis commands. This command requires two files to work, a FASTA formatted sequence file and an oligos mapping file. If you provide this command with the FASTA sequence file and oligos file provided by RTLGenomics, then you should be able to use this data in all downstream analysis.

What exactly does *No Hit* mean in my analysis?

The RTLGenomics analysis pipeline generates a number of files as discussed in the section "Analysis Archive File Descriptions" on page 25. The taxonomic information given in the "FullTaxa" and "TrimmedTaxa" are often the assigned taxonomic information, but in some cases the taxonomic information may contain the "*No Hit*" keyword for each taxonomic level. The "*No Hit*" keyword simply means that there exists no matches in our database that meet the minimum criteria to be considered likely. As such we cannot make any confident calls regarding the taxonomic classification of the OTU – even at the Kingdom/Domain level. There exists a number of reasons why this occur which we will describe below:

1. The organism's sequence is missing from our database
    a. New sequences and organisms are added to NCBI/EMBL/DDBJ on a daily basis and, while RTLGenomics makes every effort to keep our databases current, it may take a few months before new sequences are in our database.

   b. Many sequences in NCBI/EMBL/DDBJ are too short or contain no taxonomic information and are excluded from being added to our database.
2. The organism's sequence data is not yet in NCBI/EMBL/DDBJ.
   a. NCBI/EMBL/DDBJ contain a vast amount of data, however they require researchers to have already sequenced an organism before they have sequence data to provide. If no one has sequenced the organism and submitted it to those repositories, then we will not yet have the sequence.
   b. While this is not often the case, there exists the possibility that your sequence data contains an organism not yet known to science, i.e. a novel species
3. Low quality sequence
   a. While RTLGenomics does perform quality and chimera checking on your data, these algorithms are not fool-proof and low quality or chimeric sequences may have managed to make it to the taxonomic analysis stage. These sequences will often fail to identify as any organism due to their low quality or chimeric nature, causing them to be marked as "*No Hit*".

What is the difference between *Unknown* and *Unclassified* in my analysis files?

The RTLGenomics analysis pipeline generates a number of files as discussed in the section "Analysis Archive File Descriptions" on page 25. The taxonomic information given in the "FullTaxa" and "TrimmedTaxa" are often the assigned taxonomic information, but in some cases the taxonomic information may contain the "*Unknown*" and "*Unclassified*" keywords which can cause some confusion. These keywords are described as follows:

- Unknown
  - Our algorithm was unable to make a confident determination regarding the taxonomic classification at a certain level.
  - See section "USEARCH Global Search (Default)" on page 19 for more information for how we determine confidence.
- Unclassified
  - The taxonomic information retrieved from NCBI contains missing information at this level.
  - For instance, if the best match in our database is classified in NCBI down to the Family level then our database will mark the Genus and Species as "Unclassified".

How are confidence values determined?

The RTLGenomics analysis pipeline generates a number of files as discussed in the section "Analysis Archive File Descriptions" on page 25. Once each OTU has been aligned to our database, our algorithm will select the top/best six matches for the OTU and attempt to assign a confidence value to each

taxonomic level. The top match is then compared against the other five to determine the number of matches at agree with the base match at each taxonomic level. The number of agreements is then converted into a confidence value using the following equation:

$$confidence = \frac{Number\ of\ Matching\ Taxa}{Total\ Number\ of\ Taxa}$$

For instance, if OTU ID #13 has 6 top matches with the following taxa:

1) Bacteria ; Proteobacteria ; Alphaproteobacteria ; Rhizobiales ; Bradyrhizobiaceae ; Bradyrhizobium ; Bradyrhizobium sp
2) Bacteria ; Proteobacteria ; Alphaproteobacteria ; Rhizobiales ; Bradyrhizobiaceae ; Bradyrhizobium ; Bradyrhizobium sp
3) Bacteria ; Proteobacteria ; Alphaproteobacteria ; Rhizobiales ; Bradyrhizobiaceae ; Bradyrhizobium ; Bradyrhizobium sp
4) Bacteria ; Proteobacteria ; Alphaproteobacteria ; Rhizobiales ; Bradyrhizobiaceae ; Bradyrhizobium ; Bradyrhizobium japonicum
5) Bacteria ; Proteobacteria ; Alphaproteobacteria ; Rhizobiales ; Bradyrhizobiaceae ; Bradyrhizobium ; Bradyrhizobium japonicum
6) Bacteria ; Proteobacteria ; Alphaproteobacteria ; Rhizobiales ; Bradyrhizobiaceae ; Rhodopseudomonas ; Rhodopseudomonas palustris

Then each taxonomic level would receive the following confidence:

	**Kingdom**<br>Bacteria	**Phylum**<br>Proteobacteria	**Class**<br>Alphaproteobacteria	**Order**<br>Rhizobiales	**Family**<br>Bradyrhizobiaceae	**Genus**<br>Bradyrhizobium	**Species**<br>Bradyrhizobium sp
**Matching Taxa**	6	6	6	6	6	5	3
**Confidence**	1	1	1	1	1	.83	.5

Using this example, we would assign a confidence of 1 (100%) to the kingdom, phylum, class, order and family taxa. We would then assign a confidence of .83 (83%) to the genus taxon and a confidence of .5 (50%) to the species. These confidence values are then used when the “TrimmedTaxa” files are generated.

What is the difference between FullTaxa and TrimmedTaxa Files?

The RTLGenomics analysis pipeline generates a number of files as discussed in the section "Analysis Archive File Descriptions" on page 25. In our analysis data we provide most of our analysis in duplicate files, one containing the analysis using the "FullTaxa" and the other containing the analysis using the "TrimmedTaxa". Please see "How are confidence values determined?" found on page 8 as this discussion will assume you have an idea of what confidence values are and how we assign them.

The FullTaxa files are generated under the assumption that the best match is the correct one. As such each taxonomic level is assigned using that match without taking the confidence values into account. We provide this file for two reason: 1) this method for assigning taxa is similar to the method we used many years ago and we continue to provide these files for legacy purposes and 2) this helps you see what the original best match was before confidence trimming occurred. We believe these files are a powerful tool in allowing you to better get an idea of what our algorithm originally thought the data contained before confidence values were taken into account, which can help you get a better feel for the data. However, we do advise that you do not use only the FullTaxa data to perform your analysis as the lack of confidence makes the data considerably less accurate.

The TrimmedTaxa files are generated using the FullTaxa data after the confidence values have been taken into account. As such each taxonomic level is assigned only if the confidence value is greater than or equal to .51 (51%). If a taxon falls below .51, it is replaced with the "*Unknown*" keyword. Using the example data provided in the section "How are confidence values determined?" on page 8, the FullTaxa would read *Bacteria ; Proteobacteria ; Alphaproteobacteria ; Rhizobiales ; Bradyrhizobiaceae ; Bradyrhizobium ; Bradyrhizobium sp* and the TrimmedTaxa would read *Bacteria ; Proteobacteria ; Alphaproteobacteria ; Rhizobiales ; Bradyrhizobiaceae ; Bradyrhizobium ; Unknown*, where the species is now assigned "*Unknown*" due to the low confidence value.

Brief Synopsis

- FullTaxa
  - Generated under the assumption that the top/best match is completely accurate.
  - Does not take confidence values into account.
  - Provided primarily for legacy purposes and not recommended to be used for detailed analysis.
- TrimmedTaxa
  - Takes the confidence value into account at each taxonomic level.
  - Replaces low confidence taxa with the "*Unknown*" keyword.

Do my sequences contain the primer, barcodes or adapters?

Upon the completion of an order at RTLGenomics, clients will receive two zip archives containing their sequence data, the files are described in the section "File Descriptions and Formatting" starting on page 20. The raw data archive contains your sequence data directly from the sequencer with no post-processing done on our end. This data is packaged as one SFF or one pair of FASTQ files per sample. The FASTA data archive contains your sequence data after we have performed denoising and some basic quality checking on the data. This data is provided as a single FASTA formatted sequence and quality file that contains all of your sequences multiplexed together.

Please refer to the following graph in order to determine whether the primer, barcodes or adapters are on your sequences:

Data Type	Primer/Barcode	454	PGM	MiSeq[1] Single PCR	MiSeq[1] Double PCR
**Raw**	Forward Primer	Present	Present	Not Present	Present[2]
**Raw**	Reverse Primer	Not Present[3]	Not Present[3]	Not Present	Present[2]
**Raw**	Barcodes	Present[4]	Present[4]	Not Present	Not Present
**FASTA**	Forward Primer	Not Present	Not Present	Not Present	Not Present
**FASTA**	Reverse Primer	Not Present	Not Present	Not Present	Not Present
**FASTA**	Barcodes	Present[5]	Present[5]	Present[5]	Present[5]

[1] Most samples are run using the MiSeq Double PCR method, if you need to know which method your samples were run on then please contact us at lab@rtlgenomics.com.
[2] The forward primer will be found in the R1 files, the reverse primer will be found in the R2 files.
[3] The reverse primer is unlikely to be present but may be present on some reads.
[4] The barcode is present but may be hidden depending on the program used to view the reads.
[5] The barcode is present however it is not the barcode used during sequencing.

Term Definitions

Terms used within this guide are defined as follows:

- Tag
  - The term tag refers to the 8-10 bp sequence at the 5' end of the sequence read.
  - The tag is also known as the barcode in some programs.
- ASCII value
  - ASCII (American Standard Code for Information Interchange) is a character encoding scheme based on the English alphabet to encode the following: the numbers 0-9, the letters a-z, the letters A-Z, basic punctuation, control codes (such as new line), and the blank space.
  - Each letter, number and punctuation mark on a keyboard is assigned a numeric value (mostly between 0 and 127) using the ASCII table in order to create a way of encoding/decoding character symbols into computer readable digital bit patterns.

Database Maintenance Policy

RTLGenomics makes every effort to keep our databases as current as possible. This requires periodic updates to include new taxonomies and sequences as they become available. Database updates occur on an irregular schedule, so we cannot guarantee orders submitted at different times will be run against the same database version. If you have concerns about which version your samples were run against, or if you need multiple orders run against the most recent version, please contact us. Orders may be combined or rerun against a specified database for a nominal fee, however this fee may be waived if the need to merge orders is discussed at the beginning of a project.

Client Data Retention Policy

Data will made available for download (typically via a 90 day temporary link) upon completion of your project. RTL will make every reasonable effort to store all electronic data for your project for a period of 6 months from the date of notification that the project has been completed. If you have any questions regarding your data or if you need to discuss longer term storage, please contact us.

Data Analysis Methodology

Visual Overview of the Data Analysis Process

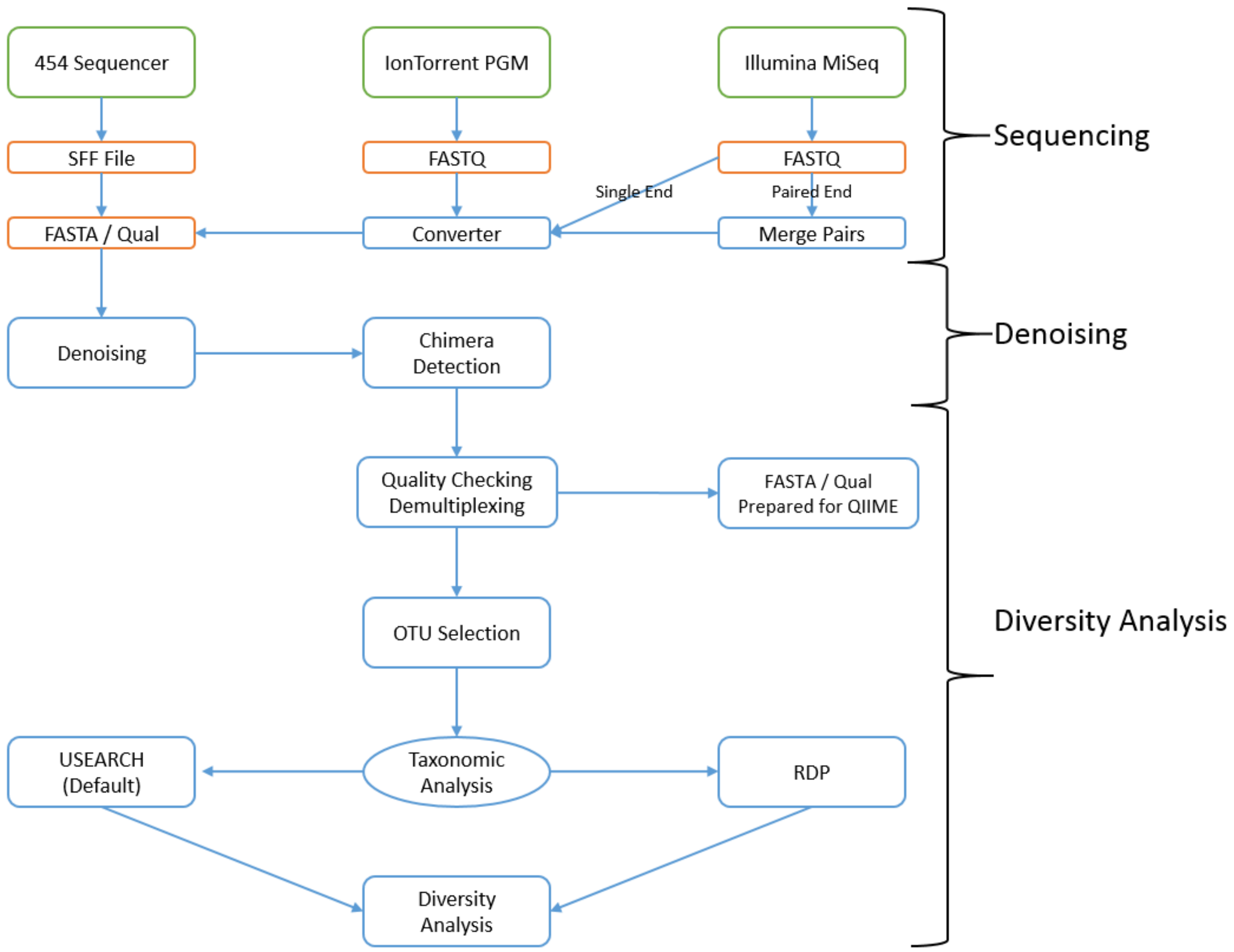

Overview of the Data Analysis Process

Once sequencing of your data has completed, the data analysis pipeline will begin processing the data. The data analysis pipeline consists of two major stages, the denoising and chimera detection stage and the microbial diversity analysis stage. During the denoising and chimera detection stage, denoising is performed using various techniques to remove short sequences, singleton sequences, and noisy reads.

With the bad reads removed, chimera detection is performed to aid in the removal of chimeric sequences. Lastly, remaining sequences are then corrected base by base to help remove noise from within each sequence. During the diversity analysis stage, each sample is run through our analysis pipeline to determine the taxonomic information for each constituent read and then this information is collected for each sample. This stage is performed for all customers whose data is sequenced using primers targeting the 16S, 18S, 23S, ITS or SSU regions. Analysis can performed on other regions but may require additional charges.

The data analysis pipeline is broken down into the following steps, each of which is discussed more thoroughly in the sections below:

- Denoising and Chimera Checking
    1. Denoising
    2. Chimera Checking
    3. SFF File Generation (454 only) – FASTQ File Generation (Ion Torrent & Illumina)
- Microbial Diversity Analysis
    1. Quality Checking and FASTA Formatted Sequence/Quality File Generation
    2. Sequence Clustering
    3. Taxonomic Identification
    4. Data Analysis

Denoising and Chimera Checking

Denoising

The process of denoising is used to correct errors in reads from next-generation sequencing technologies. According to the paper "Accuracy and quality of massively parallel DNA pyrosequencing" by Susan Huse, et al. and "Removing noise from pyrosequenced amplicons" by Christopher Quince, et al. the per base error rates from 454 pyrosequencing attain an accuracy rate of 99.5% [3] [4]. The paper "A tale of three next generation sequencing platforms: comparison of Ion Torrent, Pacific Biosciences and Illumina MiSeq sequencers" by Michael Quail, et al. states that the observed error rates generated by the Illumina MiSeq is less than .4% while the Ion Torrent PGM has an error rate of 1.78% [5]. Due to the large number of reads and even higher number of base calls per sequencing run, the total number of noisy reads can be quite substantial. In order to determine true diversity it becomes critical to determine which reads are good and which reads contain noise introduced by the experimental procedure. The RTLGenomics analysis pipeline attempts to correct this issue by denoising entire regions of data prior to performing any other steps of the pipeline.

Our analysis pipeline performs denoising by performing the following steps on each region:

1. The forward and reverse reads are taken in FASTQ format and are merged together using the PEAR Illumina paired-end read merger [6]. (Illumina MiSeq Paired End Sequencing Only)
2. The FASTQ (Illumina MiSeq and Ion Torrent PGM Only) and SFF (454 Only) formatted files are converted into FASTA formatted sequence and quality files.
3. Reads are run through an internally developed quality trimming algorithm. During this stage each read has a running average taken across the sequence and is trimmed back at the last base where the total average is greater than 25.
4. Sequence reads are then sorted by length from longest to shortest.
5. Prefix dereplication is performed using the USEARCH [7] algorithm. Prefix dereplication groups reads into clusters such that each sequence of equal or shorter length to the centroid sequence must be a 100% match to the centroid sequence for the length of the sequence. Each cluster is marked with the total number of member sequences. Sequences < 100bp in length are not written to the output file, however no minimum cluster size restriction is applied which will allow singleton clusters to exist in the output.
6. Clustering at a 4% divergence (454 & Illumina) or 6% divergence (IonTorrent) is performed using the USEARCH [7] clustering algorithm. The result of this stage is the consensus sequence from each new cluster, with each tagged to show their total number of member sequences (dereplicated + clustered). Clusters that contain <2 members (singleton clusters) are not added to the output file, thus removing them from the data set.
7. OTU Selection is performed using the UPARSE OTU selection algorithm [8] to classify the large number of clusters into OTUs.
8. Chimera checking, which is explained in more detail below in the section entitled "Chimera Checking", is performed on the selected OTUs using the UCHIME chimera detection software executed in *de novo* mode [9].
9. Each clustered centroid from step 6 listed above is then mapped to their corresponding OTUs and then marked as either Chimeric or Non-Chimeric. All Chimeric sequences are then removed.
10. Each read from step 3 is then mapped to their corresponding nonchimeric cluster using the USEARCH global alignment algorithm [7].
11. Using the consensus sequence for each centroid as a guide, each sequence in a cluster is then aligned to the consensus sequence and each base is then corrected using the following rules where C is the consensus sequence and S if the aligned sequence:
    a. If the current base pair in S is marked to be deleted, then the base is removed from the sequence if the quality score for that base is less than 30.
    b. If the current position in S is marked to have a base from C inserted, then the base is inserted into the sequence if the mean quality score from all sequences that mark the base as existing is greater than 30.

c. If the current position in S is marked as a match to C but the bases are different, then the base in S is changed if the quality score for that base is less than 30.
d. If a base was inserted or changed, the quality score for that position is updated. If the base was deleted the quality score for that position is removed.
e. Otherwise, leave the base in S alone and move to the next position.

12. The corrected sequences are then written to the output file.

Chimera Checking

As discussed in the paper "Chimeric 16S rRNA sequence formation and detection in Sanger and 454-pyrosequenced PCR amplicons" by Brian Haas, et al. the formation of chimeric sequences occurs when an aborted sequence extension is misidentified as a primer and is extended upon incorrectly in subsequent PCR cycles [10]. This can be seen in Figure 1, shown below.

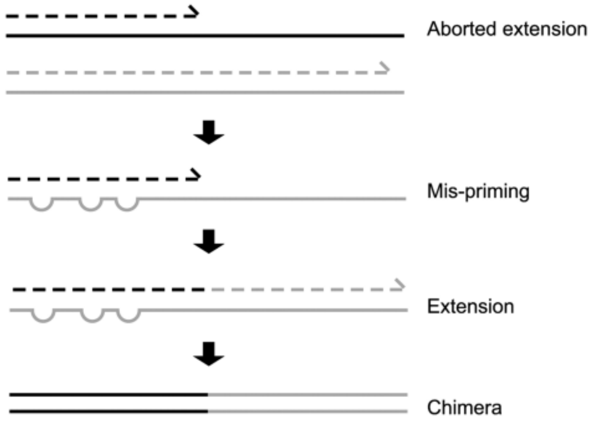

Figure 1.

Formation of chimeric sequences during PCR. An aborted extension product from an earlier cycle of PCR can function as a primer in a subsequent PCR cycle. If this aborted extension product anneals to and primes DNA synthesis from an improper template, a chimeric molecule is formed. Figure and description taken directly from "Chimeric 16S rRNA sequence formation and detection in Sanger and 454-pyrosequenced PCR amplicons" by Brian Haas, et al. **[10]**.

Because amplification produces chimeric sequences that stem from the combination of two or more original sequences [9], we will perform chimera detection using the *de novo* method built into UCHIME.

The RTLGenomics analysis pipeline performs chimera detection and removal by executing UCHIME [9] in *de novo* mode on the clustered data that was output by our denoising methods. By using this method we can determine chimeras across entire region of data even after accounting for noise and removing low quality sequences.

Microbial Diversity Analysis

In order to determine the identity of each remaining sequence, the sequences must first be quality checked and demultiplexed using the denoised data generated previously. These sequences are then

clustered into OTUs using the UPARSE [8] algorithm. The centroid sequence from each cluster is then run against either the USEARCH global alignment algorithm or the RDP Classifier against a database of high quality sequences derived from the NCBI database. The output is then analyzed using an internally developed python program that assigns taxonomic information to each sequence and then computes and writes the final analysis files.

Quality Checking and FASTA Formatted Sequence/Quality File Generation

The denoised and chimera checked reads generated during sequencing are condensed into a single FASTA formatted file such that each read contains a one line descriptor and one to many lines of sequence/quality scores. The RTLGenomics analysis pipeline takes the FASTA formatted sequence and quality files and removes any sequence which fails to meet the following quality control requirements:

1. Sequences must be at least ½ the expected length given the primer sets used.
2. Sequences must contain a valid error free barcode.

Sequences that pass the quality control screening are condensed into a single FASTA formatted sequence and quality file such that each read has a one line descriptor followed by a single line of sequence/quality data. The descriptor line in both files has been altered to contain the samples name followed by the original descriptor line, separated with a unique delimiter (::).

This stage of the pipeline creates the FASTA reads archive which contains the following files:

1. The sequence reads from all samples concatenated into a single sequence file. The original tags have been removed from each sequence and an "artificial tag" has been added in its place. The title of the file will be <name>_<order ID>.fas.
2. The quality scores from all samples concatenated into a single quality file. The scores are labeled with the corresponding sample name and will have a matching line in the .fas file. Since the original tags were removed from the sequence and an "artificial tag" was put into its place, the quality scores have been similarly altered such that the original scores for the tag have been removed and an "artificial quality tag" has been added in its place. The artificial quality tag consists of Q30s for the length of the tag. This file will be labeled <name>_<order ID>.qual.
3. A mapping file consisting of sample names included in the analysis. This file contains the information for each sample such that each line has the sample name, tag and primer used for the sample. This file will be labeled as: <name>_<order ID>.txt

OTU Selection

OTU selection clusters sequences into clusters using an OTU selection program. By default, the OTU selection method is used to determine OTUs and uses the centroid sequence for each OTU to determine taxonomic information. OTU selection is performed using the guidelines discussed in the paper "UPARSE: Highly accurate OTU sequences from microbial amplicon reads" by Robert Edgar [8]. In that paper, the following methodology is laid out in order to select OTUs:

1. Perform dereplication on the sequences.
2. Remove all singleton clusters from the data set and sort the data by abundance.
3. Trim all sequences to the same length.
4. Perform OTU clustering using UPARSE.
5. Map original reads to the OTUs

Dereplication of sequences is performed using the USEARCH prefix dereplication method [7]. Once complete we removed all singleton clusters and sorted the remaining sequences by cluster size from largest to smallest. The sequences are then run through a trimming algorithm that trims each sequence down to the same size. It should be noted that the sequences are only trimmed for UPARSE and the final taxonomic analysis is based upon the full length sequences. Next we use the UPARSE algorithm to select OTUs [8]. Using the USEARCH global alignment algorithm [7] we then assign each of the original reads back to their OTUs and write the mapping data to an OTU map and OTU table file.

Tree Building

Once OTU selection has been performed, a phylogenetic tree in Newick format is constructed. In order to construct the phylogenetic tree, multiple sequence alignment must be done on the OTU sequences in order to generate equal length aligned sequences. The multiple sequence aligner MUSCLE [11] [12], developed by Robert Edgar, is used with a maximum of 2 iterations in order to perform the alignment of the OTU data. The finished multiple sequence alignment is then passed into FastTree [13] [14], developed by Morgan Price at the Lawrence Berkeley National Lab, a program used to infer approximately-maximum-likelihood phylogenetic trees from aligned sequence data. If you would like to learn more about how FastTree works, please visit the following link: http://www.microbesonline.org/fasttree/#How.

Taxonomic Identification

In order to determine the taxonomic information for each remaining sequence, the sequences must be run through either the USEARCH global alignment program or the RDP classifier. By default the USEARCH based method is employed however the RDP classifier can be substituted if a customer has requested that we use the RDP classifier instead. In either case the data is identified using a database of

high quality sequences derived from NCBI that is maintained in house. If a customer would prefer we classify their data using a different database such as GreenGenes then we can substitute that database in place of our own. If a non-standard database is requested that requires RTLGenomics to spend time converting or creating, then a small fee may be charged.

USEARCH Global Search (Default)

The global search method uses a mixture of the USEARCH global search algorithm along with a python program to then determine the actual taxonomic assignment that is assigned to each read. This method is described in the paper "An extensible framework for optimizing classification enhances short-amplicon taxonomic assignments" by Nicholas Bokulich, et al. [15]. The paper describes a methodology in which a high quality database is used in pair with USEARCH to rapidly find the top 6 matches in the database for a given sequence. From these 6 sequences you then assign a confidence value to each taxonomic level (kingdom, phylum, class, order, family, genus and species) by taking the number of taxonomic matches that agree with the top match and then divide by the number of total matches, e.g. If Bacteria is the top kingdom match with 5 matches showing Bacteria and 1 match showing Archaea, our algorithm would assign the kingdom Bacteria a confidence of 5/6 = .83.

RDP Classifier

The RDP Classifier is naïve Bayesian classifier than can rapidly determine taxonomic information for sequences while automatically determining the confidence it has at each taxonomic level [16]. The RDP classifier is run against an internally maintained database or a customer requested database along with a taxonomic file to help determine confidence values by giving the classifier a taxonomic tree.

Diversity Analysis

Regardless of the classifier that was used, the data next enters the diversity analysis program. This program takes the OTU/Derep table output from sequence clustering along with the output generated during taxonomic identification and begins the process of generating a new OTU table with the taxonomic information tied to each cluster. This updated OTU table is then written to the output analysis folder with both the trimmed and full taxonomic information for each cluster. For each taxonomic level (kingdom, phylum, class, order, family, genus and species) four files are generated which contain the number of sequences per full taxonomic match per sample, the percentage per full taxonomic match per sample, the number of sequences per trimmed taxonomic match per sample and the percentage per trimmed taxonomic match per sample. These files are all described in more detail below.

File Descriptions and Formatting

Zip Archives

The following archives will be passed along to you upon completion of your order:

- <Name>_<OrderNumber>Raw<SequencingDate>.zip
    - This archive contains the raw FASTQ and/or raw SFF as described in the "Raw Sequence Data File Formats" section found on page 21.
- <Name>_<OrderNumber>Fasta<SequencingDate>.zip
    - This archive contains the denoised sequence data for your entire order in FASTA/Qual format as described in the "FASTA Archive File Descriptions" section found on page 23.
- <Name>_<OrderNumber>Analysis<SequencingDate>.zip
    - This archive contains the analysis data described in the "Analysis Archive File Descriptions" section found on page 25.
    - This archive will only be sent if you used a standard primer set that we have a working database for. Custom assays will likely not be analyzed.

Split Zip Archives

If any zip archive is larger than 10GB in size, we will be unable to upload the file to our file server without breaking the file into smaller chunks. In order for you to open these files you will need to download each file in the archives set (denoted with <ArchiveName>.zip.XXX where XXX is a number starting at 001 and counting upwards) and then stitch them back together prior to unzipping the archive. The following commands can be used to rebuild the zip file prior to unzipping.

Windows

Stitching the files together in Windows requires you to do the following:

1. Open a command/DOS prompt
    - In most versions of windows go to "Start menu" then type in cmd and run cmd.exe.
2. Navigate to the folder you downloaded the files into.
3. Type in the following: copy /B ArchiveName.zip.* ArchiveName.zip
4. Unzip the ArchiveName.zip file as you normally would.

Linux / Mac

Stitching the files together in Linux or Mac requires you to do the following:

1. Open a command terminal.

2. Navigate to the folder you downloaded the files into.
3. Type in the following: cat ArchiveName.zip.* > ArchiveName.zip
4. Unzip the ArchiveName.zip file as you normally would.

Raw Sequence Data File Formats

SFF File Generation

An sff file is a binary file containing detailed information regarding each read in a single file. For each read, the sff contains a flowgram, quality score and sequence with defined lengths from QC measures performed by the machine. The sff represents the raw data and includes many reads that may have been excluded due to length or chimera detection or any other filter requested for custom processing. Since the files are binary, they cannot be opened with standard text editors. Special programs like Mothur [2] or BioPython [17] are able to load their data into human readable formats and output fasta, qual, flowgram or text (sff.txt) versions. Sff files or their derivatives can then be used for further processing of the data. Sff files provided may be of two forms. In the case of an entire region containing a single investigator's samples, the entire region plus mapping file is provided. In cases where multiple investigators had samples on a single region, each sample is demultiplexed from the sff file using the Roche sffinfo tool by providing its barcode, effectively eliminating it from any read extracted. The split sff can then be used for raw data or submitted directly to archives like the NCBI's SRA. In cases where a single sff for all samples is desired but an entire quadrant is not used, an investigator may request a single sff for a nominal charge. Alternatively, it is possible to use the provided split sff files for denoising/chimera removal by modifying the mapping files. Additional instructions are available if you wish to do so.

FASTQ File Generation

FASTQ files are text based formatted data files that store the nucleotide sequences generated by the sequencer and their corresponding quality scores encoded as ASCII characters. A FASTQ file contains 4 lines per read that contain the following information:

- Line 1 contains the sequence ID (read definition) and is prepended with an "at" symbol, '@'.
- Line 2 contains the sequence data.
- Line 3 acts as a separator line between the sequence data and the quality score, it contains a single plus sign, '+'.
- Line 4 encodes the quality values for the sequence in line 2 with each quality score being represented by a single character. As such Line 2 and Line 4 must be the same length.

Decoding of the quality scores requires you to know the phred score offset that was used when the file was generated. Once you know the offset, you can take the ASCII value for the given character and

subtract the offset value to obtain the quality score. For example, if the phred offset is +33 and the character 'B' is encountered, then the quality score for that position would be 33 as 'B' is represented by the ASCII value 66 and the offset is 33 (66 – 33 = 33). Using the same logic, 'A' (represented by the value 65) would be 65-33=32 meaning the 'A' character represents a quality score of 32. A free to view ASCII table can be found here: http://www.ascii-code.com/.

Roche 454

The Roche 454 sequencers produce single SFF files for each region of a run, where runs here at RTL are broken into either 2 or 4 regions per run. Customers who opt to purchase an entire region or run of sequencing on the 454 will receive these files. Customers who opt to pay per sample will be given a single SFF file for each of their samples. SFF files that are generated one per sample will have their barcodes trimmed back by the SFF file generator. Some programs capable of reading SFF files will be able to see the original barcode and other programs will continue to ignore it. Please see the documentation for the program you are using to determine which method their software uses.

IonTorrent PGM

The IonTorrent PGM produces numerous file formats for their sequence data. We prefer to stick with the machine producing a single SFF file for the entire run that we then break down into one SFF file per sample, similar to how we run Roche 454 data. For information on what to expect when dealing with your SFF files, please see the section titled "Roche 454", found on page 22.

Illumina MiSeq

The Illumina MiSeq produces FASTQ files with a phred offset of +33. While the FASTQ file(s) generated by a MiSeq do contain all of the raw sequence data generated by the sequencer, they **do not** contain any information regarding the primer (forward or reverse). Unlike other next generation sequencing technologies, the MiSeq does not sequence the primer, instead it begins sequencing at the first base pair following the forward or reverse primer. This can make processing of your data difficult if the post processing program you decide to use requires it be able to see the primer on the sequence, however most modern programs have removed this restriction due to the prevalence of Illumina data. FASTQ files generated by the Illumina MiSeq come in two forms depending on the sequencing – either paired end or single end. Single end reads are stored in a single FASTQ file with each read in the file representing a read from the sequencer. Paired end reads, however, are slightly more complex and are covered in following section. Reads from the Illumina MiSeq are stored per sequence and are demultiplexed by the Illumina Software, thus your raw data will be missing all barcode information.

Paired End FASTQ Files

Paired end reads are stored in two FASTQ files with the first file storing the forward “half” of the read and the second file which stores the reverse “half” of the read. It should be noted that both reads are provided in forward order, meaning if you wish to link the two reads together you will first need to take the reverse complement of the reads in the second file. Depending on the insertion size and sequencing read length, the forward and reverse reads may or may not overlap at some point. Unlike other FASTQ files, the order of reads within these files must be kept in a specific order to avoid issues with most post processing programs. The reads within these two files must stay the same between the two files, meaning if you remove or move a sequence in one file, you must remove it or move it to same place in the other file to preserve the same order in both files.

Because the insert size for a paired end sequence matters, we provide two examples of how your sequences may, or may not, line up. In both examples it is assumed that you have already taken the reverse complement of the reverse reads.

Example 1 – Insert Size approx. 500bp using a 2x300 kit.

Forward---

~250 BP Forward Only | ~50 BP Alignment | ~250 BP Reverse Only

---Reverse

Example 2 – Insert Size approx. 800bp using a 2x300 kit.

Forward-----------------------------------

300 BP Forward Only | 0 BP Alignment | 300 BP Reverse Only

----------------------------------Reverse

FASTA Archive File Descriptions

The FASTA archive you receive with your data will contain the following files:

- <Name>_<OrderNumber>.fna
    - This file contains your sequence data after it has undergone quality trimming, denoising and quality checking. Each sequence in this file has had an 8 nucleotide barcode

prepended to the front of it. This barcode is not the barcode that was originally used during sequencing, instead we use a faux barcode for each sample to ensure that they each have a unique barcode.
    - **File Type:** FASTA formatted sequence data. Sequence definition lines begin with a '>' (greater than) character followed by 1 or more lines of sequence data.
    - **Recommended Program:** Any text editor.
- <Name>_<OrderNumber>.qual
    - This file contains the quality scores for your sequence data after it has undergone quality trimming, denoising and quality checking. Each quality score set in this file has had 8 fake 40 scores prepended to the front of it to account for the faux barcode added to the sequence file.
    - **File Type:** FASTA formatted sequence data. Sequence definition lines begin with a '>' (greater than) character followed by 1 or more lines of sequence quality data.
    - **Recommended Program:** Any text editor.
- <Name>_<OrderNumber>.txt
    - This file stores a table that maps each faux barcode generated in the sequence file to the sample it corresponds to. The table contains four columns and has been formatted to be compatible with QIIME, however some changes may be required if your sample names contain characters or symbols that are disallowed in QIIME.
    - The table contains the following four columns:
        - Column 1 contains the sample name (SampleID) for your sample.
        - Column 2 contains the faux barcode sequence added to you sequence file.
        - Column 3 contains the forward primer sequence that was used for that sample.
        - Column 4 contains the reverse primer sequence that was used for that sample.
        - Column 5 contains the description of the sample which is required for QIIME. Because we do not know exactly what your samples are, we instead just fill this column with the assay name.
    - **File Type:** Table written in tab-separated value (TSV) format.
    - **Recommended Program:** Any spreadsheet program or text editor.
- <Name>_<OrderNumber>.<AssayName>.oligos
    - This file stores mapping information that maps each faux barcode generated in the sequence file to the sample it corresponds to. The file contains the forward and reverse primers followed by the barcode and samples name for each sample that used that forward and reverse primer combination. This file has been formatted to be compatible with MOTHUR, however some changes may be required if your sample names contain characters or symbols that are disallowed in MOTHUR.
    - **File Type:** A standard text file.
    - **Recommended Program:** Any text editor.

Analysis Archive File Descriptions

The analysis archive you receive with your data will contain the following files:

- For each taxonomic level (<level>) where level is Kingdom, Phylum, Class, Order, Genus or Species.
    - FullTaxa.<level>.counts.txt
        - This file contains a table with the columns representing each sample in your order and the rows representing each unique taxonomic information for the top hit listed down to <level>, e.g. if <level> is Phylum then it will give each unique Kingdom/Phylum combination.
        - Each row/column intersection defines the number of sequences in the sample that matched that particular unique taxonomic information.
        - Keep in mind that the Full Taxa data shows only the taxonomic information for the top hit, regardless of what the confidence values were.
        - **File Type:** Table written in tab-separated value (TSV) format.
        - **Recommended Program:** Any spreadsheet program or text editor.
    - FullTaxa.<level>.percent.txt
        - This file contains a table with the columns representing each sample in your order and the rows representing each unique taxonomic information for the top hit listed down to <level>, e.g. if <level> is Phylum then it will give each unique Kingdom/Phylum combination.
        - Each row/column intersection defines the percent of sequences in the sample that matched that particular unique taxonomic information.
        - Keep in mind that the Full Taxa data shows only the taxonomic information for the top hit, regardless of what the confidence values were.
        - **File Type:** Table written in tab-separated value (TSV) format.
        - **Recommended Program:** Any spreadsheet program or text editor.
    - TrimmedTaxa.<level>.counts.txt
        - This file contains a table with the columns representing each sample in your order and the rows representing each unique taxonomic information for the top hit listed down to <level>, e.g. if <level> is Phylum then it will give each unique Kingdom/Phylum combination.
        - Each row/column intersection defines the number of sequences in the sample that matched that particular unique taxonomic information.
        - Keep in mind that the Trimmed Taxa data shows the taxonomic information after the confidence values are taken into account. The USEARCH method rejects the taxonomic information at a level if the confidence is below 51% while the RDPClassifier uses a minimum confidence of 80%.

        - **File Type:** Table written in tab-separated value (TSV) format.
        - **Recommended Program:** Any spreadsheet program or text editor.
    - TrimmedTaxa.<level>.percent.txt
        - This file contains a table with the columns representing each sample in your order and the rows representing each unique taxonomic information for the top hit listed down to <level>, e.g. if <level> is Phylum then it will give each unique Kingdom/Phylum combination.
        - Each row/column intersection defines the percent of sequences in the sample that matched that particular unique taxonomic information.
        - Keep in mind that the Trimmed Taxa data shows the taxonomic information after the confidence values are taken into account. The USEARCH method rejects the taxonomic information at a level if the confidence is below 51% while the RDPClassifier uses a minimum confidence of 80%.
        - **File Type:** Table written in tab-separated value (TSV) format.
        - **Recommended Program:** Any spreadsheet program or text editor.
- OTU/Derep tables
    - FullTaxa.otu_table.txt
        - This file contains a table with the columns representing each sample in your order and the rows representing each unique OTU or Dereplication Cluster. The final column contains the taxonomic information for that particular OTU/Cluster listed down to the Species level.
        - Keep in mind that the Full Taxa data shows only the taxonomic information for the top hit, regardless of what the confidence values were.
        - **File Type:** Table written in tab-separated value (TSV) format.
        - **Recommended Program:** Any spreadsheet program or text editor.
    - FullTaxa.otu_table.biom
        - This file contains a BIOM formatted copy of the OTU Table stored in FullTaxa.otu_table.txt.
        - Keep in mind that the Full Taxa data shows only the taxonomic information for the top hit, regardless of what the confidence values were.
        - **File Type:** OTU Table in BIOM Format.
        - **Recommended Program:** Any text editor or program that accepts BIOM files (e.g. QIIME).
    - TrimmedTaxa.otu_table.txt
        - This file contains a table with the columns representing each sample in your order and the rows representing each unique OTU or Dereplication Cluster. The final column contains the taxonomic information for that particular OTU/Cluster listed down to the Species level.

        - Keep in mind that the Trimmed Taxa data shows the taxonomic information after the confidence values are taken into account. The USEARCH method rejects the taxonomic information at a level if the confidence is below 51% while the RDPClassifier uses a minimum confidence of 80%.
        - **File Type:** Tables written in tab-separated value (TSV) format.
        - **Recommended Program:** Any spreadsheet program or text editor.
    - TrimmedTaxa.otu_table.biom
        - This file contains a BIOM formatted copy of the OTU Table stored in TrimmedTaxa.otu_table.txt.
        - Keep in mind that the Trimmed Taxa data shows the taxonomic information after the confidence values are taken into account. The USEARCH method rejects the taxonomic information at a level if the confidence is below 51% while the RDPClassifier uses a minimum confidence of 80%.
        - **File Type:** OTU table in BIOM format.
        - **Recommended Program:** Any Text Editor or program that accepts BIOM files (e.g. QIIME).
- **Krona Folder**
    - Raw Data Folder
        - This folder contains the raw data files that were passed to Krona in order to generate the FullTaxa and TrimmedTaxa Krona HTML files. These files were derived directly from the FullTaxa.species.counts.txt and TrimmedTaxa.species.counts.txt files. These files are provided for transparency purposes regarding how your visualization data was created.
    - FullTaxa.krona.html
        - This file contains the Krona visualization of the FullTaxa.species.count.txt file. The visualization file is a standard HTML file and should be accessible using any web browser. This visualization was generated using the FullTaxa.species.counts.txt file described before and contains data on all samples found in that file. You are able to switch between samples using the menu on the left hand side of the screen. Keep in mind that the “Collapse” checkbox is checked by default which can cause your taxonomic levels to look incorrect.
        - **This file requires an internet connection in order to work.**
        - **File Type:** HTML file with embedded code from Krona.
        - **Recommended Program:** Any Web Browser, however the developers suggest using FireFox.
    - TrimmedTaxa.krona.html

        - This file contains the Krona visualization of the FullTaxa.species.count.txt. The visualization file is a standard HTML file and should be accessible using any web browser. This visualization was generated using the TrimmedTaxa.species.counts.txt file described before and contains data on all samples found in that file. You are able to switch between samples using the menu on the left hand side of the screen. Keep in mind that the "Collapse" checkbox is checked by default which can cause your taxonomic levels to look incorrect.
        - **This file requires an internet connection in order to work.**
        - **File Type:** HTML file with embedded code from Krona.
        - **Recommended Program:** Any Web Browser, however the developers suggest using FireFox.

- **OTUs Folder**
    - OtuMap.txt
        - This file contains the mapping of each OTU identification number used in the OTU Table files. Each line contains the following information separated by tabs: the OTU identification number, the number of sequences that make up the OTU, the sequence definition for the seed sequence and then the sequence definition for each member sequence.
        - **File Type:** Table written in tab-separated value (TSV) format. The first column is the OTU #, the 2nd can be ignored, the 3rd is the OTU centroid sequence definition and columns 4+ contain each member sequence definition.
        - **Recommended Program:** Any spreadsheet program or text editor.
    - OTUs.fas
        - This file contains the OTU sequences selected during sequence clustering in fasta format. For information regarding how this file was generated please see **Error! Reference source not found.** on page **Error! Bookmark not defined.**.
        - **File Type:** FASTA formatted sequence data. Sequence definition lines begin with a '>' (greater than) character followed by 1 or more lines of sequence data.
        - **Recommended Program:** Any text editor.
- **TreeData Folder**
    - otu_map.condensed.txt
        - This file contains a condensed version of the OtuMap.txt file. Each line contains two columns separated by tabs. The first column gives the OTU identification number and the second column contains the sequence definition for the seed sequence. Thus this file contains the equivalent of columns #1 and #3 from the OTU Map.
        - **File Type:** Table written in tab-separated value (TSV) format

    - **Recommended Program:** Any Spreadsheet program or Text Editor
- otus.msa
    - This file contains the multiple sequence alignment for each OTU described in the OTU table and OTU map. This file was generated using MUSCLE as described above in the section titled Tree Building.
    - **File Type:** FASTA formatted sequence data. Sequence definition lines begin with a '>' (greater than) character followed 1 or more lines of sequence data.
    - **Recommended Program:** This file is best viewed using a MSA viewer but can also be viewed using any text editor.
- otus.tre
    - This file contains the phylogenetic tree in Newick tree format created using the otus.msa file. This file was generated using FastTree as described in the section titled Tree Building on page 18.
    - **File Type:** Newick tree formatted phylogenetic tree.
    - **Recommended Program:** This file is best viewed using a phylogenetic tree viewer but can also be viewed using any text editor.
    - **Disclaimer:** Numerous tree viewers apply restrictions on the Newick format that are not standard. As such this file may not be readable by all tree viewers. We do try to constrain the data to work with as many viewers as possible, but we can in no way make it work for all.

Recommended Programs

This section will give a brief description of some programs that we suggest you use in order to view or edit the data we have passed along.

- Text Editors
  - Let us first note that a text editor and a word processor are vastly different programs. When we say text editor we are discussing programs that often do not allow for changes in font, size or stylization. As such we strongly advise you to avoid using Microsoft Word, OpenOffice Writer, Wordpad or Google Docs to view or edit any file we provide.
  - Recommended Text Editors (Paid Usage)
    - UltraEdit: http://www.ultraedit.com/
  - Recommended Text Editors (Free)
    - Notepad++: http://notepad-plus-plus.org/
    - Unix & Linux Text Editors: GEdit / vi / emacs
- Spreadsheet Applications
  - Recommended Spreadsheet Applications (Paid Usage)
    - Microsoft Excel: http://products.office.com/en-us/excel
    - Apple Numbers: http://www.apple.com/mac/numbers/
  - Recommended Spreadsheet Applications (Free)
    - OpenOffice Calc: http://www.openoffice.org/
- Browser
  - Krona supports most browsers however the developers suggest using FireFox.
  - FireFox: https://www.mozilla.org/en-US/firefox/new/
- Tree Viewer
  - Recommended Tree Viewer (Local Installation)
    - MEGA: http://www.megasoftware.net/
  - Recommended Tree Viewer (Web Based)
    - ETE Toolkit Tree Viewer: http://etetoolkit.org/treeview/
  - For a more comprehensive list of options, see the Wikipedia article on the topic: http://en.wikipedia.org/wiki/List_of_phylogenetic_tree_visualization_software
- Multiple Sequence Alignment Viewer
  - Recommended MSA Viewer (Local Installation)
    - MEGA: http://www.megasoftware.net/
  - Recommended MSA Viewer (Web Based)
    - MView: http://www.ebi.ac.uk/Tools/msa/mview/
  - For a more comprehensive list of options, see the Wikipedia article on the topic: http://en.wikipedia.org/wiki/List_of_alignment_visualization_software

References

[1] J. G. Caporaso, J. Kuczynski, J. Stombaugh, K. Bittinger, F. D. Bushman, E. K. Costello, N. Fierer, A. G. Pena, J. K. Goodrich, J. I. Godron, G. A. Huttley, S. T. Kelley, D. Knights, J. E. Koenig, R. E. Ley, C. A. Lozupone, D. McDonald, B. D. Muegge, M. Pirrung, J. Reeder, J. R. Sevinsky, P. J. Turnbaugh, W. A. Walters, J. Widmann, T. Yatsunenko, J. Zaneveld and R. Knight, "QIIME allows analysis of high-throughput community sequencing data," *Nature Methods,* vol. 303, 2010.

[2] P. Schloss, S. L. Westcott, T. Ryabin, J. R. Hall, M. Hartmann, E. B. Hollister, R. A. Lesniewski, B. B. Oakley, D. H. Parks, C. J. Robinson, J. W. Sahl, B. Stres, G. G. Thallinger, D. J. V. Horn and C. F. Weber, "Introducing mothur: Open-source, platform-independent, community-supported software for describing and comparing microbial communities," *Appl Environ Microbiol,* vol. 75, no. 23, pp. 7537-41, 2009.

[3] S. M. Huse, J. A. Huber, H. G. Morrison, M. L. Sogin and D. M. Welch, "Accuracy and quality of massively parallel DNA pyrosequencing.," *Genome Biology,* vol. 8, no. 7, 2007.

[4] C. Quince, A. Lanzen, R. J. Davenport and P. J. Turnbaugh, "Removing Noise From Pyrosequenced Amplicons," *BMC Bioinformatics ,* vol. 12, no. 38, 2011.

[5] M. A. Quail, M. Smith, P. Coupland, T. D. Otto, S. R. Harris, T. R. Connor, A. Bertoni, H. P. Swerdlow and Y. Gu, "A tale of three next generation sequencing platforms: comparison of Ion Torrent, Pacific Biosciences and Illumina MiSeq sequencers," *BMC Genomics,* 2012.

[6] J. Zhang, K. Kobert, T. Flouri and A. Stamatakis, "PEAR: A fast and accurate Illumina Paired-End reAd mergeR," *Bioinformatics,* 2013.

[7] R. C. Edgar, "Search and clustering orders of magnitude faster than BLAST," *Bioinformatics,* pp. 1-3, 12 August 2010.

[8] R. C. Edgar, "UPARSE: highly accurate OTU sequences from microbial amplicon reads," *Nature Methods,* vol. 10, pp. 996-998, 2013.

[9] R. C. Edgar, B. J. Haas, J. C. Clemente, C. Quince and R. Knight, "UCHIME improves sensitivity and speed of chimera detection," *Oxford Journal of Bioinformatics,* vol. 27, no. 16, pp. 2194-2200, 2011.

[10] B. J. Haas, D. Gevers, A. M. Earl, M. Feldgarden, D. V. Ward, G. Giannoukos, D. Ciulla, D. Tabbaa, S. K. Highlander, E. Sodergren, B. Methé, T. Z. DeSantis, The Human Microbiome Consortium, J. F. Petrosino, R. Knight and B. W. Birren, "Chimeric 16S rRNA sequence formation and detection in Sanger and 454-pyrosequenced PCR amplicons," *Genome Research,* 2011.

[11] R. C. Edgar, "MUSCLE: multiple sequence alignment with high accuracy and high throughput," *Nucleic Acids Research,* vol. 32, no. 5, pp. 1792-1797, 2004.

[12] R. C. Edgar, "MUSCLE: a multiple sequence alignment method with reduced time and space complexity," *BMC Bioinformatics,* 2004.

[13] M. N. Price, P. S. Dehal and A. P. Arkin, "FastTree: computing large minimum evolution trees with profiles instead of a distance matrix.," *Molecular biology and evolution,* vol. 26, no. 7, pp. 1641-1650, 2009.

[14] M. N. Price, P. S. Dehal and A. P. Arkin, "FastTree 2 – Approximately Maximum-Likelihood Trees for Large Alignments," *PLOS One,* 2010.

[15] N. A. Bokulich, J. R. Rideout, K. Patnode, Z. Ellett, D. McDonald, B. Wolfe, C. F. Maurice, R. J. Dutton, P. J. Turnbaugh, R. Knight and J. G. Caporaso, "An extensible framework for optimizing classification enhances short-amplicon taxonomic assignments," *Not Yet Published,* 2014.

[16] Q. Wang, G. M. Garrity, J. M. Tiedje and J. R. Cole, "Naive Bayesian classifier for rapid assignment of rRNA sequences into the new bacterial taxonomy.," *Applied and Environmental Microbiology,* vol. 73, no. 16, pp. 5261-5267, 2007.

[17] P. J. A. Cock, T. Antao, J. T. Chang, B. A. Chapman, C. J. Cox, A. Dalke, I. Friedberg, T. Hamelryck, F. Kauff, B. Wilczynski and M. J. L. d. Hoon, "Biopython: freely available Python tools for computational molecular biology and bioinformatics," *Bioinformatics,* vol. 25, no. 11, 2009.
